# Genome-wide expression of the residual lung reacting to experimental Pneumonectomy

**DOI:** 10.1186/s12864-021-08171-3

**Published:** 2021-12-06

**Authors:** Valerio Napolioni, Fortunato Bianconi, Rossella Potenza, Francesco M. Carpi, Vienna Ludovini, Matteo Picciolini, Francesca R. Tofanetti, Antonello Bufalari, Stefano Pallotti, Camilla Poggi, Marco Anile, Niccolò Daddi, Federico Venuta, Francesco Puma, Jacopo Vannucci

**Affiliations:** 1 Genomic and Molecular Epidemiology (GAME) Lab., School of Biosciences and Veterinary Medicine, University of Camerino, Camerino, Italy; 2 Independent Researcher, Montefalco, Italy; 3 Department of Thoracic Surgery, University of Perugia Medical School, Perugia, Italy; 4 Department of Medical and Surgical Sciences (DIMEC), Alma Mater Studiorum, University of Bologna, Bologna, Italy; 5 Synbiotec S.r.l., Camerino, Italy; 6 Department of Medical Oncology, S. Maria Della Misericordia Hospital, Perugia, Italy; 7 Independent Researcher, Gubbio, Italy; 8 Department of Veterinary Medicine, University of Perugia, Perugia, Italy; 9 Genetics and Animal Breeding Group, School of Pharmacy, University of Camerino, Camerino, Italy; 10 Department of Thoracic Surgery, University of Rome Sapienza, Policlinico Umberto I, Viale del Policlinico 155, 00161 Rome, Italy

**Keywords:** Differentially expressed genes, Experimental Pneumonectomy, Genome-wide expression, Lung, Pig, Pulmonary regeneration, RNA-Seq, Translational genomics, Transcriptomics

## Abstract

**Background:**

Acute or chronic irreversible respiratory failure may occur in patients undergoing pneumonectomy. Aim of this study was to determine transcriptome expression changes after experimental pneumonectomy in swine model. Experimental left pneumonectomy was performed in five pigs under general anaesthesia. Both the resected and the remaining lung, after 60 post-operative completely uneventful days, underwent genome-wide bulk RNA-Sequencing (RNA-Seq).

**Results:**

Histological analysis showed dilation of air spaces and rupture of interalveolar septa. In addition, mild inflammation, no fibrosis, radial stretch of the bronchus, strong enlargement of airspaces and thinning of the blood supply were observed. Bioinformatic analyses of bulk RNA-Seq data identified 553 Differentially Expressed Genes (DEGs) at adjusted *P*-value below 0.001, between pre- and post-pneumonectomy. The top 10 up-regulated DEGs were *Edn1, Areg, Havcr2, Gadd45g, Depp1, Cldn4, Atf3, Myc, Gadd45b, Socs3*; the top 10 down-regulated DEGs were *Obscn, Cdkn2b,* ENSSSCG00000015738, *Prrt2, Amer1, Flrt3, Efnb2, Tox3, Znf793, Znf365.* Leveraging digital cytometry tools, no difference in cellular abundance was found between the two experimental groups, while the analysis of cell type-specific gene expression patterns highlighted a striking predominance of macrophage-specific genes among the DEGs. DAVID-based gene ontology analysis showed a significant enrichment of “Extrinsic apoptotic signaling pathway” (FDR q = 7.60 × 10^− 3^) and “Response to insulin” (FDR q = 7.60 × 10^− 3^) genes, along with an enrichment of genes involved as “Negative regulators of DDX58/IFIH1 signaling” (FDR q = 7.50 × 10^− 4^) found by querying the REACTOME pathway database. Gene network analyses indicated a general dysregulation of gene inter-connections.

**Conclusion:**

This translational genomics study highlighted the existence both of individual genes, mostly dysregulated in certain cellular populations (e.g., macrophages), and gene-networks involved in pulmonary reaction after left pneumonectomy. Their involvement in lung homeostasis is largely supported by previous studies, carried out both in humans and in other animal models (under homeostatic or disease-related conditions), that adopted candidate-gene approaches. Overall, the present findings represent a preliminary assessment for future, more focused, studies on compensatory lung adaptation, pulmonary regeneration and functional reload.

**Supplementary Information:**

The online version contains supplementary material available at 10.1186/s12864-021-08171-3.

## Background

Compensatory lung growth and alveolar regeneration have been investigated [1]; despite the great progress achieved over the past, several hypotheses remained unexplored due to the lack of advanced technological tools when originally proposed. Investigating complex biological networks by adopting system-biology approaches, leveraging high-throughput (−omics) techniques, allow to recover interesting intuitions from the past, opening the way to new research opportunities.

Pneumonectomy is associated with a decreased respiratory function, potentially leading to variable levels of oxygenation; compensatory mechanisms, hypoxia-induced, motivated studies on the vascular remodelling, empty space-filling forces, hormones, growth factors, circulating and paracrine metabolites. However, most studies focused on individual biomarkers [2–4], leaving out a systemic approach [5]; thus, a comprehensive view of lung compensatory phenomenon mechanisms is still lacking. The homeostatic reaction to respiratory failure, particularly after extended lung resection (such as pneumonectomy), remains a stimulating and challenging field of investigation. On the other hand, many aspects of the compensatory phase are known, particularly: decreased forced expiratory volume in 1 s and forced vital capacity (FEV_1_ and FVC); gas exchange tends to remain stable after compensation, but diffusion capacity tends to decrease [6].

Considering the large number of variables involved in respiratory function remodelling, an experimental approach requires pre-clinical models with high human-translational relevance. Animal models play an important role, particularly rodent and canine; swine is historically less used [7, 8]. However, swine offers the chance to perform surgical procedures like the ones applied in humans [9]. In small models, such as mice and rats, the role of the molecular reaction at the hormonal, circulatory and cellular levels have been successfully assessed [2, 10–12].

Pneumonectomy represents the main acute trigger for lung tissue growth with the aim to restore the functional loss. This biological response can be the consequence of two different mechanisms: hypertrophy and hyperplasia of the remaining tissue [13–16].

Our study aimed to determine whether and how experimental pneumonectomy in a swine model may affect transcriptional processes in the remaining contralateral lung after 60 days of mono-pulmonary breathing using bulk RNA-Seq technology and a hypothesis-free study design. Given the preliminary nature of this work, we focused on a very straightforward comparative gene expression analysis between the removed lung and the remaining one. This was followed by the estimation of cellular abundance in each bulk RNA-Seq sample using digital cytometry tools, leveraging publicly available single-cell RNA sequencing (scRNA-Seq) pig lung atlas, and analyzing cell type-specific gene expression patterns. An exploratory analysis of biological pathways (obtained through gene -ontology and -network based approaches) perturbed after experimental pneumonectomy was also performed.

## Results

### Perioperative course and histological findings

Eleven pigs underwent left pneumonectomy. All but one concluded the observation period of 60 days. This period was completely uneventful in five pigs: they had normal behaviour, food intake, growth, and wound recovery with no medical complication, fever, or signs of any disease. During daily veterinary controls, the number of respiratory acts was identical before and after operation; furthermore, no asymmetry in chest movement was observed. The remaining pigs were excluded because of perioperative adverse events.

At autopsy, chest cavity inspection showed the presence of mediastinal shift in all pigs. The right chest cavity was filled by yellowish fluid (range: 140–380 ml, median: 200 ml, mean: 226 ml), with almost complete cavity obliteration by mediastinal shifting. The posterior mediastinal pleura was open, with left lung invading the right chest cavity in 2/5 cases. Lungs showed a homogenous pink colour, becoming more whitish from the top to the bottom. Histological analysis showed a relevant variation in tissue architecture between the removed and the remaining lung.

**Fig. 1:**
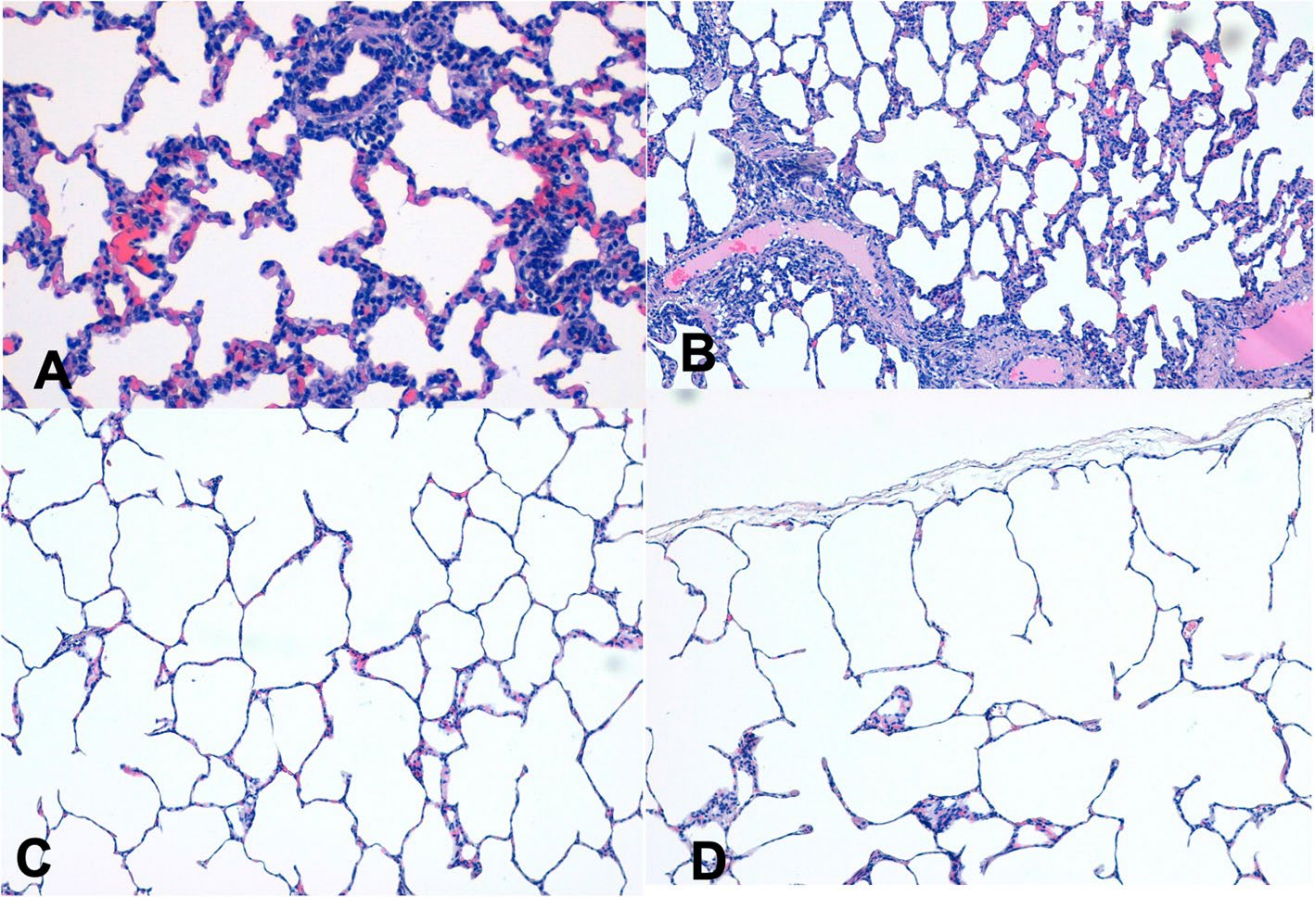
Comparative histology of the Lung. Hematoxylin-eosin staining, with 10X microscopic magnification. **A**) Normal lung after pneumonectomy, normal histology, regular ratio between airspace and blood stream, alveolar space with normal cellular components. **B**) The relationship between vessels (pink streams), air spaces and epithelial cells was maintained. **C**) Gross increase of air volume in the remaining lung, the compensatory reactions determine stretching of structural architecture of the lung, cells seem elongated and vessels lumen was restricted. **D**) The more peripheral, the more evident was the abovementioned phenomenon up to subpleural areas

At pneumonectomy, a normal ratio between airspace and blood supply was noted in the histological slides set up from the left lungs; the alveolus-capillary ratio appeared homogeneous, with normal anatomical alveolar spaces and homogeneous alveolus-capillary relationship (Figs. 1A and B). Conversely, at autopsy, right lungs showed both the rupture of the interalveolar septa and a strong increase in airspace volumes accompanied by a decrease in blood supply, with thinner and fewer capillary vessels. In some areas we also noticed a “dead space effect”. This was mostly attributable to air spaces enlargement, consistent thinning of the vascular streams, and reduction of capillary vascularization (Fig. 1C). Diffuse rupture of capillary septa was also observed, along with a significant enlargement of air spaces, particularly in the subpleural area (Fig. 1D).

**Fig. 2:**
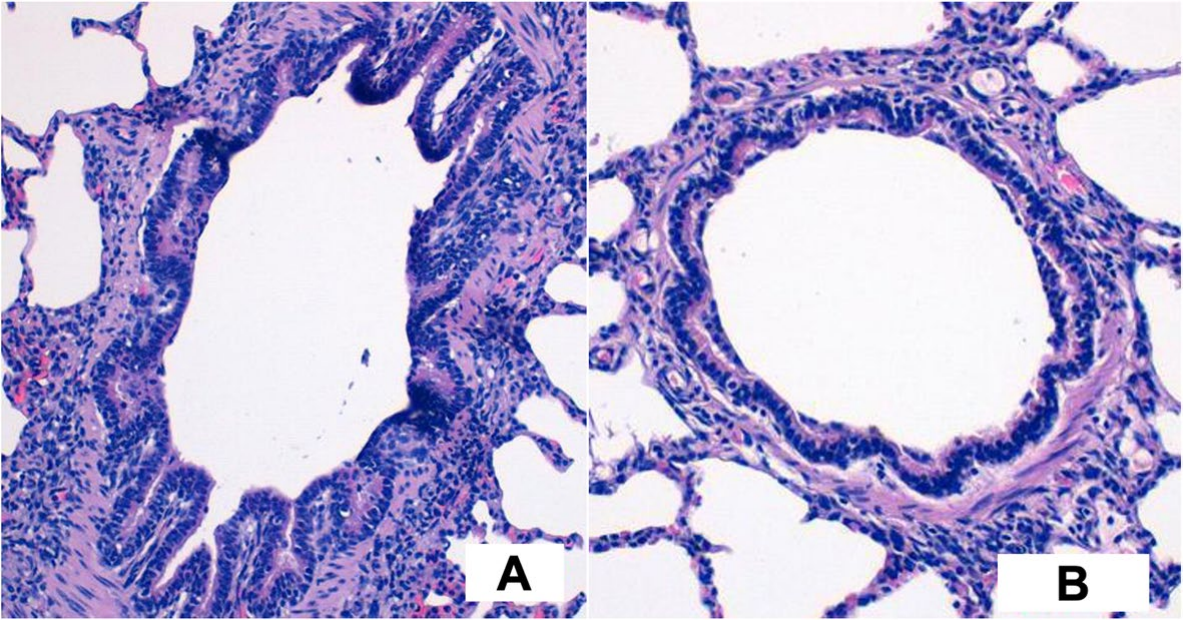
Comparative histology of the Bronchus. Hematoxylin-eosin staining, with 10X microscopic magnification. **A**) Normal histology of a peripheral bronchus, jagged epithelium with 3 to 5 rows of cylindrical cells. **B**) Reaction to pneumonectomy stretches the bronchial wall radially with thinning of epithelium and lumen enlargement

Figure 2A shows the left lung bronchial section, with sinuous and jagged margins of the bronchial epithelium (3–4-5 rows of cells). The bronchial structure was surrounded by normal lung tissue. The reaction to pneumonectomy of the remaining lung also produced bronchial effects; in particular, the wall tension and the compensatory lung volume increase led to a stretching of the bronchial wall whose margins no longer appear sinuous and jagged, but circular/ovaloid. The cellularity of the bronchial epithelium of the right lungs was reduced compared to the left lungs (Fig. 2B).

In summary, the histological analysis of the remaining lung after left pneumonectomy showed six main features:

- distal air spaces dilation, particularly in the subpleural zone;
- rupture of the interalveolar septa;
- absence of fibrosis and poor inflammation;
- dilatation of the air spaces at the periphery of the secondary lung lobules. A “vicariant emphysema” was evident;
- bronchial effect: pulmonary dilation causes a stretch of the bronchus; from a sinuous and jagged edge in the preoperative period (normal anatomical conditions) to a more cylindrical and harmonious luminal border;
- strongly increased ratio between airspaces (ventilation) and blood supply (perfusion), with evident areas of alveolar dead space.

### Bulk RNA-Seq data summary

Ten bulk RNA-seq libraries of resected lung tissue, representing the lung transcriptome before and after experimental pneumonectomy, were prepped. Using paired-end Illumina sequencing technology, 209 million raw reads were generated (20.9 Gigabases), and 202 million high-quality reads were obtained after removing low quality reads. The number of clean reads for each sample ranged from 12 to 34 million (average 20 million) (Table 1). After aligning clean reads to the reference genome (*Sscrofa11.1*/susScr11), all the samples showed an overall read mapping rate above 80.1%, and a concordant read-pair alignment rate above 74.8% (Table 1). To ensure the reliability of the analysis results, only the unique mapped reads, belonging to a concordant pair, were used for subsequent analysis.

**Table 1:** Whole transcriptome sequencing data summary

SAMPLE	PRE1	PRE3	PRE4	PRE5	PRE6	POST1	POST3	POST4	POST5	POST6
**Raw reads (2 × 100)**	22,215,114	25,366,338	28,012,764	24,912,278	35,572,116	19,130,692	14,760,032	12,1202,62	13,377,040	13,233,150
**Gigabases Sequenced**	2.2	2.5	2.8	2.5	3.6	1.9	1.5	1.2	1.3	1.3
**Left reads**										
Input	10,780,311	12,293,103	13,537,886	12,070,157	17,235,284	9,264,443	7,081,099	5,813,708	6,419,030	6,351,333
Mapped (% of input)	8,803,021(81.7%)	9,985,476 (81.2%)	11,825,297 (87.3%)	10,180,079 (84.3%)	14,637,137 (84.9%)	7,865,224 (84.9%)	5,970,220 (84.3%)	4,740,662 (81.5%)	5,229,452 (81.5%)	5,091,138 (80.2%)
Multiple alignments (% of mapped)	892,646 (10.1%)	970,380 (9.7%)	1,023,269 (8.7%)	1,033,104 (10.1%)	1,510,039 (10.3%)	801,197 (10.2%)	585,006 (9.8%)	464,993 (9.8%)	505,362 (9.7%)	505,968 (9.9%)
Multiple alignments (> 20)	5326	20,652	8678	8204	13,126	5520	4178	2699	3529	3911
**Right reads**										
Input	10,780,311	12,293,103	13,537,886	12,070,157	17,235,284	9,264,443	7,081,099	5,813,708	6,419,030	6,351,333
Mapped (% of input)	8,793,373 (81.6%)	9,968,142 (81.1%)	11,812,830 (87.3%)	10,174,749 (84.3%)	14,625,358 (84.9%)	7,853,522 (84.8%)	5,963,922 (84.2%)	4,732,430 (81.4%)	5,221,060 (81.3%)	5,080,082 (80.0%)
Multiple alignments (% of mapped)	891,840 (10.1%)	968,583 (9.7%)	1,022,056 (8.7%)	1,032,995 (10.2%)	1,509,257 (10.3%)	800,103 (10.2%)	584,803 (9.8%)	464,418 (9.8%)	505,350 (9.7%)	504,728 (9.9%)
Multiple alignments (> 20)	5326	20,659	8677	8212	13,161	5508	4195	2700	3542	3892
**Overall read mapping rate**	81.6%	81.2%	87.3%	84.3%	84.9%	84.8%	84.3%	81.5%	81.4%	80.1%
**Aligned pairs**	8,302,651	9,406,157	11,415,114	9,744,077	14,007,152	7,504,319	5,708,423	4,483,065	4,942,064	4,797,613
**Multiple alignments** (% of mapped)	843,185 (10.2%)	903,395 (9.6%)	983,803 (8.6%)	988,487 (10.1%)	1,443,293 (10.3%)	767,737 (10.2%)	559,097 (9.8%)	438,633 (9.8%)	476,211 (9.6%)	474,236 (9.9%)
**Discordant alignment** (% of mapped)	90,069 (1.1%)	116,602 (1.2%)	121,979 (1.1%)	132,889 (1.4%)	202,459 (1.4%)	93,671 (1.2%)	64,959 (1.1%)	45,215 (1.0%)	51,286 (1.0%)	49,965 (1.0%)
**Concordant pair alignment rate**	76.2%	75.6%	83.4%	79.6%	80.1%	80.0%	79.7%	76.3%	76.2%	74.8%

### Identification and analysis of differentially expressed genes (DEGs)

**Fig. 3:**
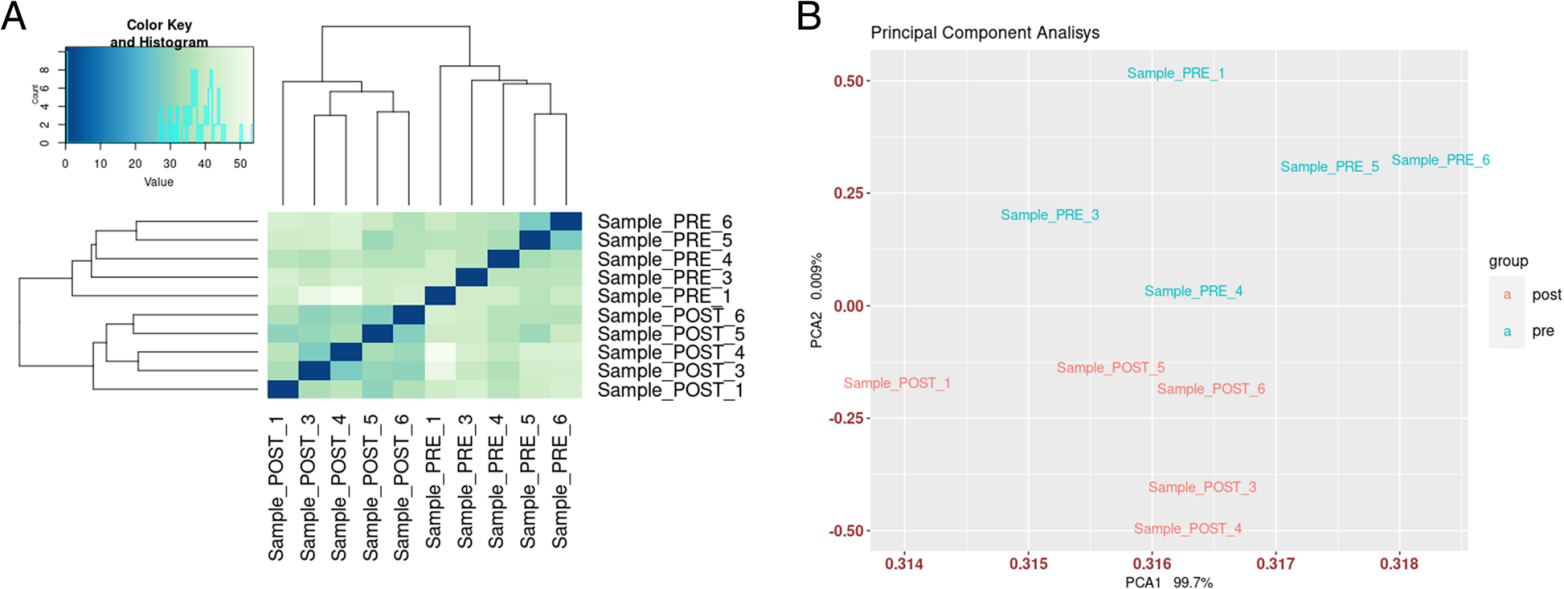
Lung transcriptome before and after experimental pneumonectomy. **A**) Heatmap of the sample-to-sample distances. Hierarchical clustering was obtained by using the Euclidean distance matrix on log2 transformation of the whole dataset**. B**) Principal component plot of the samples

A total of 21,130 genes was expressed in the ten sequenced samples, with at least one gene count reported in a unique sample, out of the total number of 25,322 annotated genes reported for the *Sscrofa11.1*/susScr11 reference genome (Additional file 1: Supplementary Table 1). The whole transcriptome of the lung, before and after experimental pneumonectomy, showed a dramatic change, as reported in Fig. 3. Indeed, both the hierarchical clustering and the principal component analysis showed a clear separation of the lung transcriptome between the pre- and post-pneumonectomy groups. A total of 553 DEGs (349 up-regulated and 204 down-regulated), at an adjusted *P*-value below 0.001, were identified. The top ten up- and down-regulated genes were reported in Table 2. The log2-fold change ranged from 2.22 to 2.74 for the up-regulated genes and from − 1.25 to − 1.59 for the down-regulated genes.

**Table 2:** Top 10 up-regulated and down-regulated gene between the pre- and post-pneumonectomy groups. P=P-value; P-adj = FDR-adjusted P-value

Group	Gene_name	Description	baseMean	log2FoldChange	P	P-adj
Up-regulated	*Edn1*	Endothelin 1	905.52	2.74	3.18E-35	6.66E-32
	*Areg*	Amphiregulin	30.26	2.68	2.35E-16	8.60E-14
	*Havcr2*	Hepatitis A virus cellular receptor 2	472.48	2.66	4.67E-54	2.94E-50
	*Gadd45g*	Growth arrest and DNA damage inducible gamma	811.90	2.65	2.90E-22	2.03E-19
	*Depp1*	DEPP1 autophagy regulator	310.97	2.38	1.95E-27	2.46E-24
	*Cldn4*	Claudin 4	2971.36	2.36	4.72E-21	3.12E-18
	*Atf3*	Activating transcription factor 3	1434.89	2.34	2.24E-23	2.17E-20
	*Myc*	MYC proto-oncogene, bHLH transcription factor	1118.21	2.26	1.39E-37	3.50E-34
	*Gadd45b*	Growth arrest and DNA damage inducible beta	1294.38	2.26	4.03E-57	5.07E-53
	*Socs3*	Suppressor of cytokine signaling 3	161.60	2.22	2.42E-22	1.79E-19
Down-regulated	*Obscn*	Obscurin, cytoskeletal calmodulin and titin-interacting RhoGEF	98.40	−1.59	4.07E-13	8.40E-11
	*Cdkn2b*	Cyclin dependent kinase inhibitor 2B	79.18	−1.46	2.06E-09	2.01E-07
	ENSSSCG00000015738	n.a	48.35	−1.34	4.92E-08	3.44E-06
	*Prrt2*	Proline rich transmembrane protein 2	88.19	−1.32	1.78E-06	7.44E-05
	*Amer1*	APC membrane recruitment protein 1	57.88	−1.32	1.94E-08	1.52E-06
	*Flrt3*	Fibronectin leucine rich transmembrane protein 3	274.64	−1.30	2.86E-07	1.61E-05
	*Efnb2*	Ephrin B2	371.29	−1.29	8.55E-07	4.25E-05
	*Tox3*	TOX high mobility group box family member 3	47.38	−1.27	1.52E-07	9.27E-06
	*Znf793*	Zinc finger protein 793	36.04	−1.25	3.45E-07	1.88E-05
	*Znf365*	Zinc finger protein 365	57.98	−1.25	1.84E-07	1.10E-05

### Cellular abundance and cell type-specific gene expression patterns

**Fig. 4:**
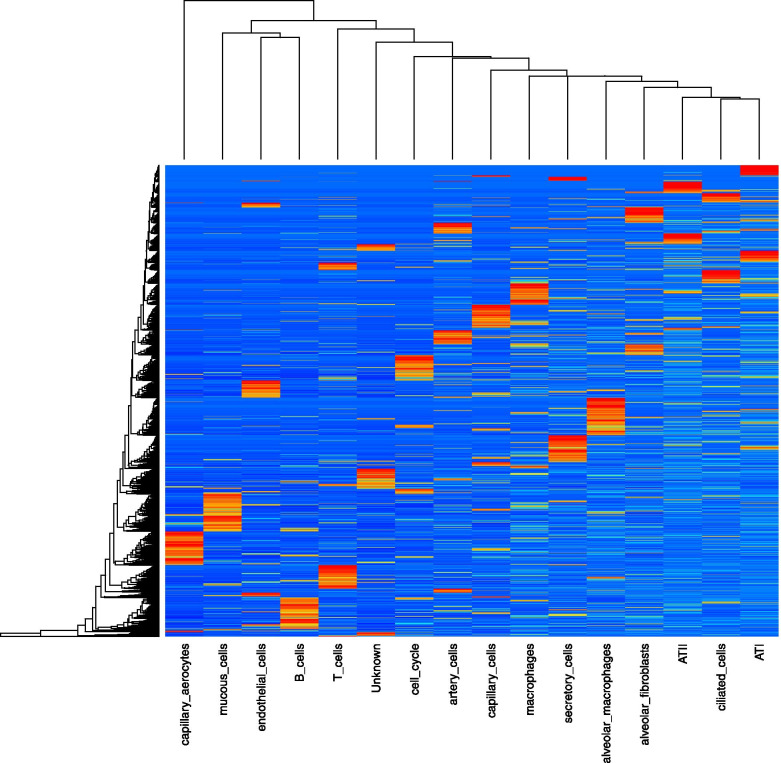
Custom signature matrix based on the reference scRNA-Seq dataset [17] obtained from CIBERSORTx [18]

**Fig. 5:**
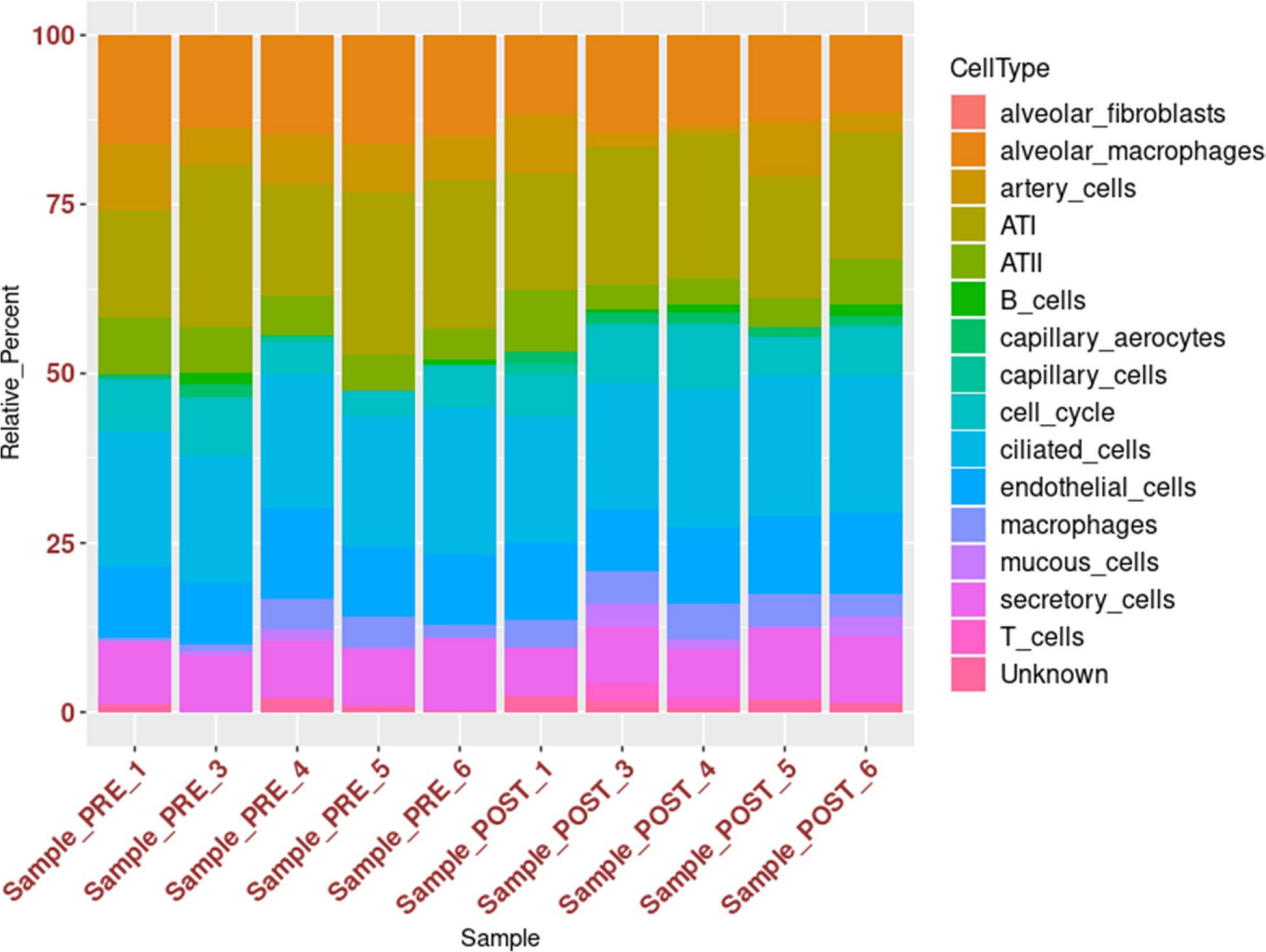
Estimated cellular composition obtained from CIBERSORTx [18]. Differences in estimated cell type fractions between pre- and post-pneumonectomy samples were tested using Kruskal-Wallis test (all *P* > 0.345)

Leveraging the single-cell RNA sequencing (scRNA-Seq) dataset of adult pig lung obtained from the study of Zhang et al. [17], encompassing 15 different cell types {Alveolar epithelial type 1 (ATI), Alveolar epithelial type 2 (ATII), alveolar fibroblasts, endothelial cells, ciliated cells, capillary cells, capillary aerocytes, artery cells, mucous cells, secretory cells, macrophages, alveolar macrophages, T cells, B cells and cell cycle-mitotic state status}, the cellular fractions composing each of the ten bulk RNA-Seq samples, were determined using CIBERSORTx [18]. First, a custom signature matrix based on the reference scRNA-Seq dataset [17], that captured all major cell subsets considered, was established (Fig. 4). Thus, the scRNA-Seq-derived signature matrix was applied to resolve cellular composition of the ten bulk RNA-Seq samples from the pre- and post- pneumectomy groups. As shown in Fig. 5, the most represented cell types were ATI and ciliated cells (mean, 19.8%), alveolar macrophages (mean, 13.9%), endothelial cells (mean, 10.9%), secretory cells (mean, 8.8%), cell cycle-mitotic state status (mean, 6.6%), artery cells (mean, 6.0%), ATII (mean, 5.9%) and macrophages (mean, 3.5%); the other seven cell types considered, including the “unknown”, were absent (alveolar fibroblasts) or present at very low percentages (less or equal to 1%). No statistically significant difference (by Kruskal-Wallis test) in estimated cell type fractions, between pre- and post- pneumectomy samples, was found. This evidence points to a lack of bias in the determination of DEGs from bulk RNA-Seq samples attributable to differences in cellular composition.

The imputation of cell type-specific gene expression profiles was restricted to the nine most represented cell-types (mean cellular type ≥3.5%). 295 out of the 553 DEGs identified between pre- and post-pneumectomy groups were uniquely attributed to a cell type (Additional file 2: Supplementary Table 2). Notably, their majority (213/295, 72.2%) was attributable to macrophages, followed by ATII (28/295, 9.5%), secretory cells (25/295, 8.5%), cell cycle-mitotic state (20/295, 6.8%), ATI (7/295, 2.4%) and endothelial cells (2/295, 0.7%). No cell-type specific DEG was detected for ciliated cells, alveolar macrophages, and artery cells.

### Gene ontology functional analysis and interaction networks

To get a better insight into the biological pathways perturbed by experimental pneumonectomy, a functional annotation analysis was performed, using DAVID Bioinformatics Resources [19], searching for significantly enriched genes and associated pathways. With this purpose, we considered only the 553 DEGs. Gene ontology analysis revealed a significant enrichment of “Extrinsic apoptotic signaling pathway” (FDR q = 7.60 × 10^− 3^) and “Response to insulin” (FDR q = 7.60 × 10^− 3^) genes, and of “Negative regulators of DDX58/IFIH1 signaling” (FDR q = 7.50 × 10^− 4^) from the REACTOME pathway database. No significant KEGG pathway was found.

**Fig. 6:**
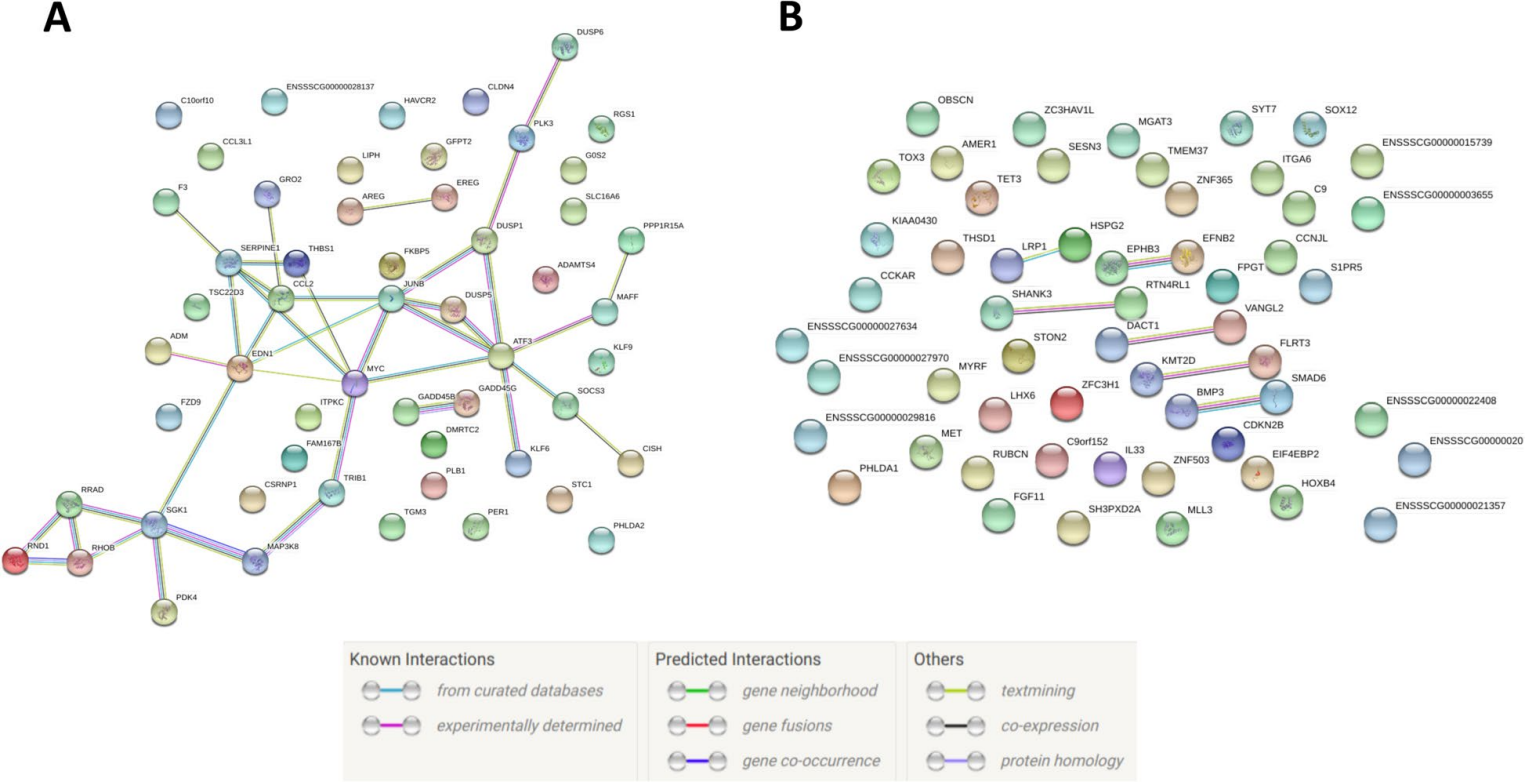
Biological networks of Differentially Expressed Genes (DEGs) **A**) Up-regulated genes. **B**) Down-regulated genes

**Fig. 7:**
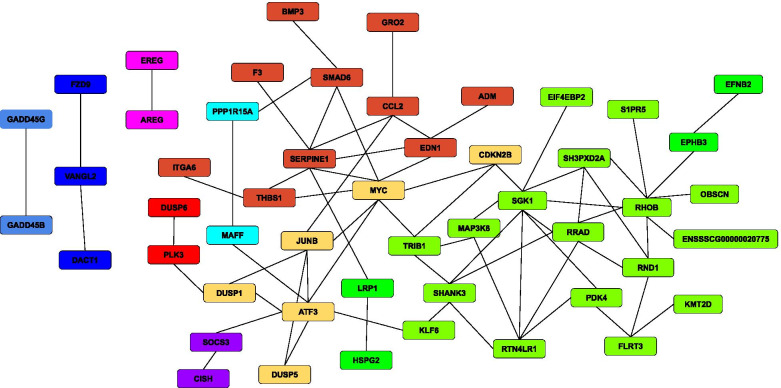
Reconstruction of the whole biological network using ModuLand [20]. Network analysis was performed considering the entire set of genes that belong to both the upper and lower tail of the fold change distribution

Then, considering the genes falling in the lower and upper tail of the distribution of the fold change, the corresponding interaction networks were inferred (Additional file 3: Supplementary Table 3, Fig. 6, Fig. 7). Interestingly, network-structures in the above-cited figures turned out very different from each other. Since the network in Fig. 6 contained a greater number of highly interconnected nodes, most of the regulatory mechanisms active before the pneumonectomy were completely dysregulated after the resection. Moreover, Table 3 shows the results of the pathway enrichment analysis using the nodes reported in Fig. 1. Finally, applying ModuLand [20], 11 modules were identified based on the network reconstructed using the entire set of genes belonging to both the upper and lower tail of the fold change distribution (Fig. 7).

**Table 3:** Pathway enrichment analysis. FDR = False Discovery Rate

Pathway description	Observed genes	FDR p-value	Matching proteins
TNF signaling pathway	6	4.03e-05	CCL2, EDN1, GRO2, JUNB, MAP3K8, SOCS3
MAPK signaling pathway	6	0.00187	DUSP1, DUSP5, GADD45B, GADD45G, MAP3K8, MYC
p53 signaling pathway	4	0.00187	GADD45B, GADD45G, SERPINE1, THBS1
FoxO signaling pathway	4	0.0139	GADD45B, GADD45G, PLK3, SGK1
Hippo signaling pathway	4	0.0139	AREG, FZD9, MYC, SERPINE1
ErbB signaling pathway	3	0.0447	AREG, EREG, MYC

## Discussion

Several studies assessed signals and mechanisms of compensatory lung growth [1, 2, 4]. Nevertheless, the biological bases underlying compensatory and regenerative processes after pneumonectomy remain largely unknown [21]. The present study aimed to define the genome-wide transcriptional response of swine lung to experimental pneumonectomy.

Our results highlighted a relevant transcriptional response of the remaining lung compared to the resected one, with approximatively 2% of the annotated *Sscrofa11.1*/susScr11 reference genome being Differentially Expressed Genes (DEGs). Indeed, of the 25,322 annotated genes, 21,130 were found to be expressed in the swine lung (with at least one gene count reported in a unique sample). Notably, digital cytometry analysis did not evidence any significant difference in cell type composition between pre- and post-pneumectomy, supporting the goodness and reliability of DEGs analysis carried out on the bulk-RNA samples.

Despite the lack of significant difference in cell fractions between the two experimental groups, the analysis of cell type-specific gene expression patterns showed a striking predominance of macrophage-specific genes among the DEGs. Thus, it is conceivable that macrophages underwent a relevant transcriptional remodeling after pneumectomy. This is in line with mounting evidence supporting a model in which macrophages play essential roles in the regeneration of organs, including limbs, intestines, liver, kidney, and heart [22–31]. As matter of fact, Lechner et al. [32] recently demonstrated that macrophages play a key role in lung adaptation/compensatory growth following pneumectomy in mice; they found that interstitial macrophages are required for regeneration in post-subtotal lung resection, since macrophages are a component of the regenerative type 2 alveolar epithelial stem cells (AEC2s) niche. Indeed, it has been suggested that lung compensatory growth after pneumectomy requires the coordinated proliferation and rearrangement of numerous epithelial and stromal cell types, including AEC2s, an epithelial stem cell population capable of self-renewal and differentiation into type 1 alveolar epithelial cells [32].

The top-ten of up-regulated and down-regulated DEGs between the pre- and post-pneumonectomy groups (Table 2) showed the presence of 6 genes, all up-regulated, namely, *Areg, Edn1, Gadd45b, Gadd45g, Myc* and *Socs3,* contributing to each of the six significantly enriched pathways (Table 3), targeting TNF, MAPK, p53, FoxO, Hippo and ErbB signaling, respectively. All these pathways were linked to hypoxia, tissue regeneration, and tumor genesis.

TNF signaling pathway is initiated in response to cellular stress and inflammatory signals, leading to the activation of pro-apoptotic pathways and cytokine cascades [33]. *Edn1* drives the activation and proliferation of fibroblast cells and their differentiation into myofibroblasts leading to collagen deposition. Its expression in lung tissue is increased in idiopathic pulmonary fibrosis [34, 35]. However, in our model no fibrosis was observed, potentially due to the length of observation time; moreover, *Edn1* is also a potent vasoconstrictor peptide, and its dysregulation has been implicated in coronary microvascular dysfunction, non-small cell lung cancer development and progression [36, 37]. *Socs3* gene regulates the lung inflammatory response, promoting pulmonary injury repair through the inhibition of JAK2/STAT3, leading to a reduced expression of inflammatory factors [38–40], while contributing to the protection of lung endothelium [41].

The activation of MAPK, p53, and FOXO signaling pathways in the lung mesenchyme is crucial for lung development as these pathways regulate different cellular functions (e.g., proliferation, differentiation, and apoptosis) in response to an endogenous or exogenous stress [42–44]. A crucial gene, acting as modulator in all the three mentioned pathways, is *Gadd45b* which has been involved in response reactions to cellular damage and lung carcinogenesis [45, 46]. Similarly, *Gadd45g*, as a member of the DNA damage-inducible gene family inhibiting cell growth in response to stress shock and induces apoptosis [47], acts as tumor suppressor gene frequently inactivated epigenetically in multiple tumors [47, 48]. High expression of *Myc* gene occurs in proliferating and dividing cells during development and in adult tissues [49–51]. Particularly, *Myc* expression coordinates a broad variety of crucial processes for lung tissue regeneration [52]. In humans, the dysregulation of this oncogene was observed in several tumors, including lung cancer [53].

Hippo signaling, another pathway found to be enriched in our study, plays crucial roles in surfactant homeostasis and coordination of peripheral lung differentiation [54].

ErbB is an essential multiple regulatory pathway both in normal physiology and in cancer [55, 56]. Beside the *Myc* gene, we found the top upregulated *Areg* gene in this enriched pathway. *Areg* leads to airway remodeling following lung transplantation [57] and promotes the airway inflammatory recovery response [58]. The gene is also strongly expressed by alveolar macrophage in lipopolysaccharide-induced acute lung injury [59].

Several top up-regulated genes were not included in the significantly enriched pathways. These genes were shown to be involved in reactions against pulmonary injury and lung metabolism, thus representing interesting candidates for future investigations. *Depp1* was reported to be an autophagy-related hypoxia-responsive gene [60]. *Cldn4* encodes a transmembrane protein of the alveolar-capillary membrane [61], expressed by the epithelial cells of the whole respiratory system [62], acting as paracellular permeability regulator during alveolar fluid clearance [63]. Moreover, *Cldn4* is also involved in resolution of pulmonary edema [64], while being used as indicator of pulmonary damage [63–65]. Finally, *Atf3* promotes cellular growth, invasion, and collagen synthesis, while inhibiting apoptosis, playing a crucial role in the lung, as demonstrated by its association with protection against acute pulmonary injury and viral infection [66, 67].

Interestingly, none of the top 10 down-regulated genes was included in the enriched pathways. These genes seem to have important tissue structural roles. Indeed, *Obscn* encodes giant cytoskeletal proteins expressed in a wide variety of cell types, mostly in cardiac and other striated muscles where it contributes to cellular process with structural and regulatory roles and myofibrils organization [68–70]. *Cdkn2b* regulates critical processes for lung regeneration such as extracellular matrix remodeling, endothelial proliferation, and cell cycle progression [71–73]. *Amer1* is a widespread expressed gene during mouse embryonic development which acts as a negative modulator of WNT/β-catenin pathway serving pleiotropic functions during organogenesis [74]. *Flrt3* is expressed in a wide variety of tissues and is involved in cell adhesion and adipocytokine signaling pathways [75], while, notably, being reported as the most downregulated gene in thoracic visceral adipose tissue after lung allograft reperfusion [76]. *Efnb2* is an angiogenesis factor regulating both pulmonary branch and vascular development through the control of VEGF-induced angiogenesis and lymphangiogenesis [77]. In addition, an increased expression of *Efnb2* has been shown to promote fetal lung development in rats [78]. On the other hand, reduced gene expression inhibits alveolar development while intranasal administration of the protein inhibits the apoptosis process in the alveolar epithelial cells [79]. Also, *Efnb2* inactivation results in distal lung dysplasia, alveolar crest formation and reduced distal lung compliance [80]. *Prrt2*, *Tox3* and *RNA-Znf793* genes, respectively, were found to be linked to different neuronal functions or to immune-related mechanisms involved in the immune response to lung solicitations [81–85]. Thus, it is likely that their role may be linked to a general biological response to dramatic insults/modifications, rather than representing lung-specific mechanisms underlying compensatory and regenerative processes after pneumonectomy. Nonetheless, the strong dysregulation in the expression of those genes after experimental pneumonectomy should not be disregarded since they may be still playing a crucial role in the regenerative processes after the surgical procedure.

The exploratory nature of this study necessarily needs to be acknowledged; indeed, we aimed to provide the first genome-wide expression study occurring after experimental pneumonectomy in a swine model. Nonetheless, this study presented several limitations. The hypothesis-free design did not provide the possibility for a final comprehensive answer to any specific question and analyses the different gene expression in a single lung biopsy, although at the same site. The experiment would have been more interesting if there were different gene signatures in different locations. The experimental model is well-known but the reaction to pneumonectomy has been reported to display species-specific peculiarities [4]. The number of tested models (sample size = 5 × 2) was quite limited. However, our experimental approach guaranteed a highly controlled environment, under standardized procedures, minimizing the presence of experimental biases.

## Conclusions

Compensatory lung reaction after pneumonectomy is a fascinating research field. The histological assessment showed that air space tended to expand in terms of volume with a relative decrease of the blood flow. This anatomical change was twinned to a series of provoked gene patterns that were identified, pinpointing to a strong involvement of the macrophage’s cellular component. The exploratory nature of this research might open to further, more focused investigations.

Currently, there is no possible claim for a real translation into clinical practice. However, from a clinical point of view, the progressive increase of end stage lung disease will require a series of treatment to slow the functional loss. In this regard, considering the limits of organ transplants and the prevalence of chronic pulmonary diseases, more focused applied research on the genetic involvement in the compensation after organ failure might be a chance to create new treatment strategies. This study provides the first general sight on several genes and pathways possibly playing a role in the process. It represents a very preliminary panel with several interesting potentials. The possibility to handle the decrease or increase of function of an organ by stimulating a gene or a pool of genes is still missing in the clinical scenario. This ability would be surprisingly revolutionary for those diseases that currently just receive supportive and symptomatic therapies. The most updated technologies have given new options to investigate elusive and complex phenomenon like the alveologenesis. The application of these technologies could open new research opportunities with the futuristic goal to modulate the expression of key genes whose malfunction will lead to organ insufficiency.

## Materials and methods

### Ethical statement

The animal care protocol and the study design were approved by the institutional committee for laboratory animal welfare of the University of Perugia and by the Italian Ministry of Health, centrally dedicated authority. The research was performed according to the most updated recommendations on animal manipulation. All surgical procedures were carried out in accordance with Local and Central Authorities for Animal care, following the “Guide for the Care and Use of Laboratory Animals” [86].

### Animal housing and surgery

A total of 11 female Landrace x large White pigs (*Sus scrofa domesticus*), 39 ± 4 kg was transferred to the experimental centre 5 days before surgery and adapted to the new environmental condition. They were fed once a day and had water ad libitum. All animals underwent left pneumonectomy under general anaesthesia and orotracheal intubation. Each pig was approached in right lateral decubitus and the left lung was removed through thoracotomy. The whole procedure and the perioperative management were performed according to the previously described technique [87, 88]. After surgery, pigs were observed for 60 days, then they were painlessly sacrificed under general anaesthesia and the right lung was harvested.

### Experimental design

This study was hypothesis-free. It aimed at showing both the anatomical and gene expression differences between two lungs after 60 days of single lung breathing. The removed lung and the remaining lung after pneumonectomy were compared at autopsy and microscopically assessed. The functional reaction after pneumonectomy was investigated using a comparative RNA-Seq of the lung biopsy specimens. Out of the total series of pigs undergoing pneumonectomy, RNA-Seq was performed only for those animals that showed a completely smooth post-operative course (*N* = 5). Animals with records of signs of any kind of post-operative events (fever after post-operative day 2, cough, prostration, lack of appetite, etc.), even if minimal, were excluded from the study to eliminate every potentially misleading expression of genes. Soon after pneumonectomy (left lung) and soon after euthanasia (right lung), samples of pulmonary tissue were harvested from the same area of the organs (dorsal segment of caudal lobe) and stored in cryovials at − 80 °C. The dorsal segment of the caudal lobe was chosen because is central, easy to recognize in harvested lungs at bench, anatomical variants are rare, and it is vertical to the hilum. Sampling was performed at the half of the distance between visceral pleura and hilum. Other samples from both lungs were microscopically assessed. Slides with hematoxylin-eosin staining of homologous zones belonging to corresponding segments were comparatively assessed to evaluate the morphological differences (air space volume, blood supply, number of vessels, distance from vessels to vessels, vessels caliber, distance between septa, width of epithelial cells layers, radial stretching, cellularity). The comparative histological analysis was only descriptive and performed by an independent pathologist who read left lung slides (before) and right lung slides (afterwards), pig-by-pig. The pathologist was asked to report a comparative difference if there was a delta of at least 30% in the findings between the two samples. No quantitative assessment of the morphological changes was performed.

### RNA isolation, library construction and sequencing

Total RNA was extracted from frozen tissues after thawing and homogenizing by IKA Ultra-Turrax and QIAzol Lysis Reagent. RNA was extracted with the phenol chloroform method. From the aqueous phase, RNA was automatically purified by BioRobot EZ1using EZ1 RNA Universal Tissue kit instrument according to the manufacturer’s instructions (Qiagen S.p.A., Milan, Italy). RNA was eluted in 50 μl of RNase-free water and stored at − 80 °C until use. The total RNA extracted from the samples was prepped according to the Illumina TruSeq RNA Sample prep kit. Thus, each sample was indexed, pooled by 10, and sequenced on a single lane of Illumina Flow Cell PE v3 (Illumina Inc., CA, USA). Sequencing was performed on Illumina HiSeq1500 with TruSeq SBS chemistry (200 cycles), generating 2 × 100 Paired-Ends reads.

### Raw sequencing data processing and reads alignment

Raw sequencing data were processed using CASAVA v1.8 and the bioinformatic analyses were performed through the Bioconductor package on R. Quality control was carried out with FastQC [89]. Per-sequence and per-base analyses were carried out to filter reads according to the following criteria: sequence-read distribution = 75 bp, 100% coverage in all bases, GC-content ~ 50%, ~ 25% of A, T, G and C nucleotide contributions, ambiguous base-content < 0.1% and a Phred score higher than 30 (i.e., base-calling accuracy larger than 99.9%). Short sequence reads were assembled, mapped, and annotated by using as template the most recent pig reference genome (*Sscrofa11.1*/susScr11). Read count matrix was obtained by counting the number of reads mapping on specific gene according to the gene set for each sample. Multimap reads were discarded.

### Differentially expressed genes (DEGs) analysis

Data were normalized by a regularized-logarithm transformation. A Principal Component Analysis (PCA) was conducted to determine samples similarity between the two conditions. DEGs were identified using DESeq2 package [90], by applying the *DESeq* function, and treating the two groups, pre- and post-pneumonectomy, as unpaired, with no covariate adjustment. The resulting *p*-values were adjusted through the Bonferroni correction method and a threshold was used to select the most statistically significant genes (*p* < 0.001). Then, the resulting genes were sorted according to their fold change value.

### Digital Cytometry from bulk RNA-Seq data and identification of cell type-specific gene expression patterns

The estimation of cellular abundance and cell type-specific gene expression patterns from bulk RNA-Seq data were performed using scSorter [91] and CIBERSORTx [18]. scSorter was used to label each cell included in the single-cell RNA (scRNA) sequencing dataset of adult pig lung [17] considered as reference, according to the 38 cell-specific expression markers reported by Zhang et al. [17]. CIBERSORTx [18] allowed the creation of the scRNA-Seq signature matrix upon which the cell fractions and the cell type-specific gene expression patterns were imputed from bulk RNA-Seq data. Differences in imputed cell fractions between pre- and post-pneumectomy groups were tested by Kruskal-Wallis test, setting the level of statistical significance at *P* < 0.05 (two-sided).

### Gene ontology functional analysis and interaction networks

Gene ontology and interaction network analyses were performed on the top up- and down- regulated genes, selected by computing the 10th and 90th percentile of the fold change distribution across the full set of expressed genes. These two subsets of genes were used to reconstruct both the up- and down- regulated interaction networks, by querying the String database, and selecting “*Sus scrofa*” as reference organism to retrieve the information about the interactions [92]. Since String is a protein-protein association network database, it automatically mapped input genes into the associated proteins. Functional annotation analysis was performed using DAVID Bioinformatics Resources, using the entire “*Sus scrofa*” as gene background [19]. ModuLand algorithm [20] was used to identify clusters of nodes in the networks. ModuLand is implemented as a Cytoscape plug-in, an open-source bioinformatics platform for the analysis of experimental data and the integration of biomolecular network models [93]. Moreover, this algorithm returns key nodes bridging two or multiple modules and predicting the function of the whole module.

## Supplementary Information

**Additional file 1: Supplementary Table 1.** List of the 21,130 genes expressed in the ten sequenced samples out of the total number of 25,322 annotated genes reported for the *Sscrofa11.1*/susScr11 reference genome.

**Additional file 2: Supplementary Table 2.** List of DEGs uniquely attributable to a single cell-type.

**Additional file 3: Supplementary Table 3.** Selection of expressed genes falling in the lower and upper tail of the distribution of the fold change.

## Data Availability

The dataset analysed in the current study is available in the SRA (NCBI) repository (BioSample accession: SAMN17840146) https://www.ncbi.nlm.nih.gov/bioproject/700846

## Abbreviations

DEGs: Differentially Expressed Genes
PCA: Principal component analysis
Edn1: Endothelin 1
Areg: Amphiregulin
Havcr2: Hepatitis A virus cellular receptor 2
Gadd45g: Growth arrest and DNA damage inducible gamma
Depp1: DEPP1 autophagy regulator
Cldn4: Claudin 4
Atf3: Activating transcription factor 3
Myc: MYC proto-oncogene, bHLH transcription factor
Gadd45b: Growth arrest and DNA damage inducible beta
Socs3: Suppressor of cytokine signaling 3
Obscn: Obscurin, cytoskeletal calmodulin and titin-interacting RhoGEF
Cdkn2b: Cyclin dependent kinase inhibitor 2B
Prrt2: Proline rich transmembrane protein 2
Amer1: APC membrane recruitment protein 1
Flrt3: Fibronectin leucine rich transmembrane protein 3
Efnb2: Ephrin B2
Tox3: TOX high mobility group box family member 3
Znf793: Zinc finger protein 793
Znf365: Zinc finger protein 365
